# Supplementary material for: Seeing the forest for the coral trees: involvement and perceptions of reef threats among coral restoration volunteers in Roatan, Honduras
Source: Camb Prism Coast Futur. 2024 Oct 29;2:e14. doi: 10.1017/cft.2024.13 (PMC12337603; doi:10.1017/cft.2024.13)
Supplement: Garcia and Rivera supplementary material [file S2754720524000131sup001.zip › atlas-ti-data-import-description.pdf]

Appendix 3: Data Preparation and Import into Atlas.Ti

1. Exporting the Google Sheet with all survey response data
    a. Follow-up responses were matched to original respondents in same rows
        - Renamed all questions from the follow up survey to begin with "FOLLOW UP:"
    b. Sheet with all data combined renamed MasterDoc (to keep separate from initial survey/follow up surveys)
    c. Duplicated MasterDoc
    d. Exported as .xlsx
2. Organizing the Data Within Atlas.Ti
    a. Atlas.ti automatically generated document groups that corresponded to each of the survey questions
        - Manually combined the document groups created for each of the Spanish survey questions with the equivalent document groups in English, with comments detailing which were merged
        - Excluded email addresses and the final three survey questions (see Appendix 1) from the import
    b. Manually corrected errors in individual responses and made comments about each correction on its Atlas.ti document:

Survey Question	Response Error	P#	Justification/ Correction
How many lifetime dives have you completed?	Responded "None" instead of "60 or more"	47	Responded "60 or more" to question "How many dives have you completed **in the Bay Islands** (Roatan, Utila, Guanaja)", which necessitates the same response for lifetime dives; changed response and document group in Atlas.ti
Which dive shop are you taking the course with?	Responded "."	47	Participant was in BIRR restoration course, offered only at Subway Watersports.<br><br>Added to correct document group in Atlas.ti.
When did you first participate in coral restoration? Please select your best guess if unsure.	Input invalid date format	10, 18	Verbally confirmed correct dates and changed document groups within Atlas.ti
Which best describes your previous	Responded "*I have never participated in coral restoration*	6, 49, 75	Confirmed each respondent's participation in and completion of coral restoration courses through RMP and BIRR databases.

experience with coral restoration?	*before and don't plan to in the immediate future*” instead of “*I will participate in coral restoration for the first time today*” (or “*Nunca participé en la restauración de arrecifes de coral”* instead of “*Voy a participar en la restauración de arrecifes de coral por la primera vez hoy.*”)		In Atlas.Ti, manually corrected and noted document group change.
	Responded “*I will participate in coral restoration for the first time today*” instead of “*I have never participated in coral restoration before and don't plan to in the immediate future*”	59, 61	Confirmed no participation in coral restoration by either respondent at the dive shops indicated.
	Responded “*I have previously participated in coral restoration, in Roatan or in other places*” instead of “*I have never participated in coral restoration before and don't plan to in the immediate future*”	19	Verbally confirmed that respondent’s prior experience was not coral restoration as the survey intended it to be understood.
	Responded “*I have previously participated in coral restoration, in Roatan or in other places*” instead of “*I will participate in coral restoration for the first time today*”	44	Date when the restoration course was taken was listed as the first time participating in coral restoration; must be a date prior to the survey and restoration course.
What coral restoration course are you affiliated with?	Responded “*PADI Distinctive Specialty Course in Coral Restoration*” instead of “*RMP Coral Restoration Ambassador*.”	27, 74	Course indicated was not offered at the dive shops selected in the survey.
	Responded “*RMP Coral Restoration Ambassador*” instead of “*PADI Distinctive Specialty Course in Coral Restoration.*”	45	Course indicated was not offered at the dive shop selected in the survey.
FOLLOW UP: Which best describes your previous experience with coral restoration?	Responded “*I have never participated in coral restoration before and don't plan to in the immediate future*” instead of “*I have previously participated in coral restoration, in Roatan or in other places”*	6, 9, 12, 37, 52, 66	Indicated in their initial surveys either, “I will participate in coral restoration for the first time today,” or “I have previously participated in coral restoration, in Roatan or in other places.”

c. Renamed the document groups (formerly survey questions) as follows:

Survey Question	Atlas.Ti Header Relabel for Document Groups
How many lifetime dives have you completed?	LD
How many dives have you completed **<u>in the Bay Islands</u>** (Roatan, Utila, Guanaja)?	LDBI
Which best describes your previous experience with coral restoration?	RE:0 (*control divers*)<br>RE:1 (*restoration learners*)<br>RE:2 (*experienced restoration divers*)
What coral restoration course are you affiliated with?	Course: BIRR<br>Course: RMP<br>Course: RMP
Which business(es) do you dive with?	Shop: RD (*Roatan Divers)*<br>Shop: CTD (*Coconut Tree Divers*)<br>Shop: Sun Divers<br>Shop: RG (*Reef Gliders*)<br>Shop: Subway Watersports
How many total dives have you completed for coral restoration?	*After reviewing answers to this question, responses were subdivided into two restoration experience levels:*<br>RE:2.1 (*up to 6 dives*)<br>RE:2.2 (*more than 6 dives*)
In what location(s) have you participated in coral restoration?	RestorationLocation: Bay Islands Only<br>RestorationLocation: Bay Islands + Other<br>RestorationLocation: Not Bay Islands
When did you first participate in coral restoration? Please select your best guess if unsure.	Since: 2022<br>Since: 2021<br>Since: 2020 or before
List any actions you think **<u>individuals</u>** or **<u>communities</u>** could take that would help protect the health of coral reefs. See definition above. If you're not sure, it's OK to write your best guess or "I don't know."	Q1
List any actions you think **<u>governments</u>** could take that would help protect the health of coral reefs. If you're not sure, it's OK to write your best guess or "I don't know."	Q2
[How much do you agree with the following: "I do a lot to protect the health of coral reefs."]	LQ1-LQ6 (*1 equivalent to "strongly disagree", 6 equivalent to "strongly agree")*
In what ways, if any, do you intend to protect the health of coral reefs in the future? Please include anything you already do and intend to continue doing.	Q3
What is your gender?	Gen
What is your age?	Age

What is your country or region of origin?	Origin::Honduras<br>Origin::Other Latin America<br>Origin:: USA<br>Origin:: Canada<br>Origin::Europe<br>Origin: India
Where do you currently live?	LivesIn::Honduras<br>LivesIn::Other Latin America<br>LivesIn:: USA<br>LivesIn:: Canada<br>LivesIn::Europe

   d. Renamed all document groups from follow-up survey questions with the same conventions described above, with FU (“follow-up”) preceding the name (e.g., “FULD” for “follow-up lifetime dives”)

3. Preparing data for qualitative analysis
   a. Translated Spanish responses to English using ChatGPT3.5 (very lightly edited for clarity/improved translation as needed)
   b. Coded survey responses with identified ways to protect coral reef health:
      - Codes applied at the sentence level where possible
