# Supplementary material for: Seeing the forest for the coral trees: involvement and perceptions of reef threats among coral restoration volunteers in Roatan, Honduras
Source: Camb Prism Coast Futur. 2024 Oct 29;2:e14. doi: 10.1017/cft.2024.13 (PMC12337603; doi:10.1017/cft.2024.13)
Supplement: Garcia and Rivera supplementary material [file S2754720524000131sup001.zip › blank-surveys-english-spanish.pdf]

Coral Survey

Thank you for taking the time to help us learn more about attitudes towards coral reefs! Your responses are valuable. This survey takes about 5 minutes to complete.

* Indicates required question

1. Email *

2. I consent to the data I've entered here being used for research. This information will be anonymized and analyzed in aggregate. If I am a minor, my legal parent(s) or guardian(s) have consented as well. *

Mark only one oval.

- Yes

Lifetime Dives

3. How many lifetime dives have you completed? *

Mark only one oval.

- None
- 1-14
- 15-59
- 60 or more

4. How many dives have you completed **in the Bay Islands** (Roatan, Utila, Guanaja)? *

Mark only one oval.

- None
- 1-14
- 15-59
- 60 or more

5. Which best describes your previous experience with coral restoration? *

Mark only one oval.

- I have never participated in coral restoration before and don't plan to in the immediate future. *Skip to question 6*
- I will participate in coral restoration for the first time today. *Skip to question 7*
- I have previously participated in coral restoration, in Roatan or in other places. *Skip to question 9*

6. Which business(es) do you dive with? *

Mark only one oval.

- Roatan Divers
- Reef Gliders
- Coconut Tree Divers
- Sun Divers
- Subway Watersports
- Other: ______________________

Skip to question 12

Course Info

7. What coral restoration course are you affiliated with? *

Mark only one oval.

- ( ) RMP Coral Restoration Adventurer
- ( ) RMP Coral Restoration Ambassador
- ( ) PADI Distinctive Specialty Course in Coral Restoration
- ( ) Utila Coral Foundation
- ( ) Other: ______________________

8. Which dive shop are you taking the course with? *

Mark only one oval.

- ( ) Roatan Divers
- ( ) Reef Gliders
- ( ) Coconut Tree Divers
- ( ) Sun Divers
- ( ) Subway Watersports
- ( ) Other: ______________________

Skip to question 12

Restoration Experience

9. How many total dives have you completed for coral restoration?

____

10. In what location(s) have you participated in coral restoration?

____

11. When did you first participate in coral restoration? Please select your best guess if unsure.

____

Example: January 7, 2019

Knowledge and Attitudes

For this survey, a healthy coral reef is defined as one with a high amount of living coral, large and diverse populations of marine life, and the ability to support human communities.

12. List any actions you think **individuals** or **communities** could take that would help protect the health of coral reefs. See definition above. If you're not sure, it's OK to write your best guess or "I don't know." *

13. List any actions you think **governments** could take that would help protect the health of coral reefs. If you're not sure, it's OK to write your best guess or "I don't know." *

14. *

Mark only one oval per row.

	Strongly disagree	Disagree	Somewhat disagree	Somewhat agree	Agree	Strongly agree
**How much do you agree with the following: "I do a lot to protect the health of coral reefs."**	⬭	⬭	⬭	⬭	⬭	⬭

15. In what ways, if any, do you intend to protect the health of coral reefs in the future? Please include anything you already do and intend to continue doing. *

____

____

____

____

____

Demographics and Submission

16. What is your gender?

Mark only one oval.

⬭ Male

⬭ Female

⬭ Other: ____________________

17. What is your age?

Mark only one oval.

- 17 or younger
- 18-29
- 30-41
- 42-53
- 54-65
- 66 or older

18. What is your country or region of origin?

Mark only one oval.

- USA
- Canada
- Europe
- Honduras
- Other Latin American (not Honduras)
- Other: ______________________

19. Where do you currently live?

Mark only one oval.

- USA
- Canada
- Europe
- Honduras
- Other Latin American (not Honduras)
- Other: ______________________

20. Are you willing to complete a brief follow-up survey in the coming month? *

Mark only one oval.

- Yes
- No
- Other: ____________________

21. Are you willing to answer more questions about coral restoration in a follow up interview within the next two weeks? If you select "yes," you may be contacted via the email provided for this survey. *

Mark only one oval.

- Yes
- No
- Other: ____________________

22. Is there anything else you'd like to add?

This content is neither created nor endorsed by Google.

Google Forms

Encuesta sobre Arrecifes de Coral

¡Gracias por tomarse el tiempo para ayudarnos a aprender más sobre las actitudes hacia los arrecifes de coral! Sus respuestas son valiosas. Esta encuesta tarda unos 5 minutos en completarse.

* Indicates required question

1. Email *

____

2. Doy mi consentimiento para que los datos que he ingresado aquí se utilicen para la investigación. Esta información será anonimizada y analizada en conjunto. Si soy menor de edad, mis padres o tutores legales también han dado su consentimiento. *

Mark only one oval.

- Sí

Inmersiones

3. ¿Cuántas veces en la vida ha buceado con scuba? *

Mark only one oval.

- Ninguna vez
- 1-14
- 15-59
- 60 o más

4. ¿Cuántas veces en la vida ha buceado **en las Islas de la Bahía** (Roatán, Utila, Guanaja)? *

Mark only one oval.

- Ninguna vez
- 1-14
- 15-59
- 60 o más

5. Describa sus experiencias ayudando con la restauración de arrecifes de coral. *

Mark only one oval.

- Nunca participé en la restauración de arrecifes de coral. *Skip to question 6*
- Voy a participar en la restauración de arrecifes de coral por la primera vez hoy. *Skip to question 7*
- He participado al mínimo una vez antes en la restauración de arrecifes de coral. *Skip to question 9*

6. ¿Con cuál empresa normalmente bucea? *

Mark only one oval.

- Roatan Divers
- Reef Gliders
- Coconut Tree
- Sun Divers
- Subway Watersports
- Other: ____________________

Skip to question 12

El curso

7. ¿Cuál curso de restauración hace? *

Mark only one oval.

- RMP Coral Restoration Adventurer
- RMP Coral Restoration Ambassador
- PADI Distinctive Specialty Course in Coral Restoration
- Utila Coral Foundation
- Other: __________

8. ¿Con cuál empresa de buceo hace el curso? *

Mark only one oval.

- Roatan Divers
- Reef Gliders
- Coconut Tree
- Sun Divers
- Subway Watersports
- Other: __________

Skip to question 12

Experiencias con Restauración de Arrecifes de Coral

9. ¿Cuántas inmersiones en total ha realizado para la restauración de coral? *

____

10. ¿En qué ubicación(es) ha buceado para participar en la restauración de coral? *

____

11. ¿Cuándo fue la primera vez que usted participó en la restauración de coral? Si no está seguro, escoge una fecha probable.

____

Example: January 7, 2019

Conocimiento y Actitúd

Para esta encuesta, un arrecife de coral saludable se define como uno con una gran cantidad de corales vivos, poblaciones grandes y diversas de vida marina, y la capacidad de sostener a las comunidades humanas.

12. Haga una lista de cualquieras acciones que se le ocurran sobre que podrían hacer **individuos** o **comunidades** para proteger la salud de los arrecifes de coral. (Por favor revise la definición de arriba.) Si no está seguro, puede escribir su mejor suposición o "No lo sé". *

13. Haga una lista de cualquieras acciones que se le ocurran sobre que podrían hacer **gobiernos** para proteger la salud de los arrecifes de coral. (Por favor revise la definición de arriba.) Si no está seguro, puede escribir su mejor suposición o "No lo sé". *

14. *

Mark only one oval per row.

	Definitivamente en desacuerdo	En desacuerdo	Más o menos en desacuerdo	Más o menos de acuerdo	De acuerdo	Definitivamente de acuerdo
**Está de acuerdo?: "Hago mucho para proteger la salud de los arrecifes de coral."**	⬭	⬭	⬭	⬭	⬭	⬭

15. ¿Qué acciones, si haya algunas, tiene intención de hacer en el futuro para proteger la salud de los arrecifes de coral? Debe incluir acciones que ya hace si tiene intención de seguir haciéndolas en el futuro. *

Demografía (ultima sección)

16. Usted es:

Mark only one oval.

- ⬭ Hombre
- ⬭ Mujer
- ⬭ Other: ____________

17. ¿Cuántos años tiene?

Mark only one oval.

- 17 o menos
- 18-29
- 30-41
- 42-53
- 54-65
- 66 o más

18. ¿Cuál es su país/region de origen?

Mark only one oval.

- Honduras
- Otro país latinoamericano (no Honduras)
- USA
- Canada
- Europa
- Other: ____________________

19. ¿En qué país/region vive ahora?

Mark only one oval.

- Honduras
- Otro país latinoamericano (no Honduras)
- USA
- Canada
- Europa
- Other: ____________________

20. ¿Está dispuesto a completar una breve encuesta de seguimiento el próximo mes? *

Mark only one oval.

- Sí
- No
- Other: ______________________

21. ¿Está dispuesto a hacer una entrevista para contestar mas preguntas acerca de la restauración de corales en las próximas dos semanas? Si respondió que "sí," es posible que establezcamos contacto consigo pronto con el mismo email que usted usó para llenar este formulario. *

Mark only one oval.

- Sí
- No
- Other: ______________________

22. Si tiene algún otro comentario, por favor escríbalo abajo.

____
____
____
____
____

This content is neither created nor endorsed by Google.

Google Forms

Coral + Divers Follow-Up Survey

Thank you for taking the time to help us learn more about attitudes towards coral reefs! Your responses are valuable. This survey takes about 5 minutes to complete.

* Indicates required question

1. Email *

2. I consent to the data I've entered here being used for research. This information will be anonymized and analyzed in aggregate. If I am a minor, my legal parent(s) or guardian(s) have consented as well. *

Mark only one oval.

- Yes

Lifetime Dives

3. How many lifetime dives have you completed? *

Mark only one oval.

- None
- 1-14
- 15-59
- 60 or more

4. How many dives have you completed **in the Bay Islands** (Roatan, Utila, Guanaja)? *

Mark only one oval.

- None
- 1-14
- 15-59
- 60 or more

5. Which best describes your previous experience with coral restoration? *

Mark only one oval.

- I have never participated in coral restoration before and don't plan to in the immediate future. *Skip to question 8*
- I have previously participated in coral restoration, in Roatan or in other places. *Skip to question 6*

Restoration Experience

6. How many total dives have you completed for coral restoration?

____

7. In what location(s) have you participated in coral restoration?

____

Knowledge and Attitudes

For this survey, a healthy coral reef is defined as one with a high amount of living coral, large and diverse populations of marine life, and the ability to support human communities.

8. List any actions you think **individuals** or **communities** could take that would help protect the health of coral reefs. See definition above. If you're not sure, it's OK to write your best guess or "I don't know." *

9. List any actions you think **governments** could take that would help protect the health of coral reefs. If you're not sure, it's OK to write your best guess or "I don't know." *

10. *

Mark only one oval per row.

	Strongly disagree	Disagree	Somewhat disagree	Somewhat agree	Agree	Strongly agree
**How much do you agree with the following: "I do a lot to protect the health of coral reefs."**	⬭	⬭	⬭	⬭	⬭	⬭

11. In what ways, if any, have you helped protect the health of coral reefs in the last year? *

Demographics and Submission

12. Where do you currently live?

Mark only one oval.

- USA
- Canada
- Europe
- Honduras
- Other Latin American (not Honduras)
- Other: ____________________

13. Would you like to be entered to receive a complimentary hat or t-shirt from Roatan Marine Park or Bay Islands Reef Restoration for your participation in this survey? If you select "yes" and are one of the participants selected, you will receive a follow-up email in the coming month to confirm your best shipping address.

Mark only one oval.

- Yes, I'd like to be considered for a complimentary hat/t-shirt
- No, I do not want to receive a complimentary hat/t--shirt

14. Is there anything else you'd like to add?

This content is neither created nor endorsed by Google.

Google Forms

Encuesta de seguimiento sobre los buzos y arrecifes de coral

¡Gracias por tomarse el tiempo para ayudarnos a aprender más sobre las actitudes hacia los arrecifes de coral! Sus respuestas son valiosas. Esta encuesta tarda unos 5 minutos en completarse.

* Indicates required question

1. Email *

____

2. Doy mi consentimiento para que los datos que he ingresado aquí se utilicen para la investigación. Esta información será anonimizada y analizada en conjunto. Si soy menor de edad, mis padres o tutores legales también han dado su consentimiento. *

Mark only one oval.

- ◯ Sí

Inmersiones

3. ¿Cuántas veces en la vida ha buceado con scuba? *

Mark only one oval.

- ◯ Ninguna vez
- ◯ 1-14
- ◯ 15-59
- ◯ 60 o más

4. ¿Cuántas veces en la vida ha buceado **en las Islas de la Bahía** (Roatán, Utila, Guanaja)? *

Mark only one oval.

- Ninguna vez
- 1-14
- 15-59
- 60 o más

5. Describa sus experiencias ayudando con la restauración de arrecifes de coral. *

Mark only one oval.

- Nunca participé en la restauración de arrecifes de coral. *Skip to question 9*
- He participado al mínimo una vez antes en la restauración de arrecifes de coral. *Skip to question 6*

Experiencias con Restauración de Arrecifes de Coral

6. ¿Cuántas inmersiones en total ha realizado para la restauración de coral? *

____

7. ¿En qué ubicación(es) ha buceado para participar en la restauración de coral? *

____

8. ¿Cuándo fue la primera vez que usted participó en la restauración de coral? Si no está seguro, escoge una fecha probable.

____

Example: January 7, 2019

Conocimiento y Actitúd

Para esta encuesta, un arrecife de coral saludable se define como uno con una gran cantidad de corales vivos, poblaciones grandes y diversas de vida marina, y la capacidad de sostener a las comunidades humanas.

9. Haga una lista de cualquieras acciones que se le ocurran sobre que podrían hacer **individuos** o **comunidades** para proteger la salud de los arrecifes de coral. (Por favor revise la definición de arriba.) Si no está seguro, puede escribir su mejor suposición o "No lo sé". *

10. Haga una lista de cualquieras acciones que se le ocurran sobre que podrían hacer **gobiernos** para proteger la salud de los arrecifes de coral. (Por favor revise la definición de arriba.) Si no está seguro, puede escribir su mejor suposición o "No lo sé". *

11. *

Mark only one oval per row.

	Definitivamente en desacuerdo	En desacuerdo	Más o menos en desacuerdo	Más o menos de acuerdo	De acuerdo	Definitivamente de acuerdo
**Está de acuerdo?: "Hago mucho para proteger la salud de los arrecifes de coral."**	◯	◯	◯	◯	◯	◯

12. ¿Qué acciones, si haya algunas, tomaste en el último año para proteger la salud de los arrecifes de coral? *

Ultima sección

13. ¿En qué país/region vive ahora?

Mark only one oval.

- Honduras
- Otro país latinoamericano (no Honduras)
- USA
- Canada
- Europa
- Other: ____________

14. Si tiene algún otro comentario, por favor escríbalo abajo.

This content is neither created nor endorsed by Google.

Google Forms
