# Supplementary material for: Seeing the forest for the coral trees: involvement and perceptions of reef threats among coral restoration volunteers in Roatan, Honduras
Source: Camb Prism Coast Futur. 2024 Oct 29;2:e14. doi: 10.1017/cft.2024.13 (PMC12337603; doi:10.1017/cft.2024.13)
Supplement: Garcia and Rivera supplementary material [file S2754720524000131sup001.zip › semi-structured-interview-consent-forms-english-spanish.pdf]

Consent to Use of Semi-Structured Interview Responses for Research

* Indicates required question

1. Write your full name below. *

2. I am the person listed above, and I consent to my responses in this semi-structured interview being recorded and analyzed for research on coral restoration and reef threat perceptions that Sierra Garcia is conducting in collaboration with the Roatan Marine Park, Bay Islands Reef Restoration, and Coral Reef Alliance. Once identifying data such as my name is removed, I may be quoted anonymously in reports to the above organizations, or in peer-reviewed published research. *

   Mark only one oval.

   ◯ Yes

3. Today's Date

   Example: January 7, 2019

4. Email *

This content is neither created nor endorsed by Google.

Google Forms

Consentimiento para el uso de respuestas de entrevistas semiestructuradas para investigación

* Indicates required question

1. Escriba sus nombres y apellidos. *

2. Soy la persona mencionada y doy mi consentimiento para que mis respuestas en esta entrevista semi-estructurada sean grabadas y analizadas para la investigación sobre la restauración de corales y las percepciones de amenazas a los arrecifes. Esta investigación está manejado por Sierra Garcia, en colaboración con el Parque Marino de Roatán, Bay Islands Reef Restoration y Coral Reef Alliance. Después de que se eliminen los datos personales, como mi nombre, se me puede citar de forma anónima en resúmenes para las organizaciones mencionadas anteriormente o en investigaciones publicadas revisadas por pares científicos. *

   Mark only one oval.

   ◯ Sí

3. Fecha de hoy

   Example: January 7, 2019

4. Correo electrónico (email) *

This content is neither created nor endorsed by Google.

Google Forms
