# Supplementary material for: Seeing the forest for the coral trees: involvement and perceptions of reef threats among coral restoration volunteers in Roatan, Honduras
Source: Camb Prism Coast Futur. 2024 Oct 29;2:e14. doi: 10.1017/cft.2024.13 (PMC12337603; doi:10.1017/cft.2024.13)
Supplement: Garcia and Rivera supplementary material [file S2754720524000131sup001.zip › semi-structured-interview-protocol-english-spanish.docx]

**Appendix 2: Semi-Structured Interview Protocol**

*Goal:* Gain additional context for the cognitive, affective, and behavioral engagement in coral reef health/threats/solutions for people involved in coral restoration.

*Before:*

- Offer compensation of 250 lempiras (roughly equivalent to USD$10) to dive professionals who agree to interview
- Confirm the individual has taken the initial research survey and completed the semi-structured interview consent form in the language
- Have their survey responses at hand to reference
- Schedule time and space for uninterrupted meeting
- Read the following intro script (or equivalent in Spanish):
  - Thanks for taking the time to do this interview! Your answers will be very valuable for understanding how divers like yourself think about coral reef health. I’m Sierra, and I’m collaborating with the Roatan Marine Park, Coral Reef Alliance, and Bay Islands Reef Restoration on this research. This interview will be anonymized, so anything you share won’t be linked to details that identify you. Can I record this conversation? *[pause for response]* Thanks! Are you ready to start?

*During:*

- Modulate body language + tone for consistency between interviews
- Avoid interrupting or intervening if they’re making incorrect statements
- Ask questions in order written unless interviewee brings up a topic that makes a different question more natural to ask first

**Appendix 2: Semi-Structured Interview Questions (English)**

- What feelings do you associate with the future of coral reefs? (1)
- What feelings do you associate with coral restoration? (2)
  - *How would you describe coral restoration to other people in your life that haven’t participated in it?*
- In the survey, you answered “Y” in response to “I do a lot to protect the health of coral reefs.” Can you tell me why you gave yourself that answer? (3)
  - *How are you protecting coral reef health?*
  - *What actions to protect reef health would you like to take more?*
  - *What could help people like you more easily take those actions?*
- On the survey you wrote ‘X’ as a way to protect coral reef health. Can you tell me more about why you chose that? (4)
  - *Can you describe what [X] means?*
  - *Can you tell me how/why that protects the health of reefs?*
  - *Any additional answers come to mind now?*
- What have your biggest takeaways been from [taking/teaching/participating in] the coral restoration theory on dry land? (5)
- What have your biggest takeaways been from [taking/teaching/participating in] the coral restoration dives? (6)
  - *If teaches restoration: What do you hope the biggest takeaways from the course are for the divers you teach?*
- Is there anything else you’d like to add?

**Appendix 2: Semi-Structured Interview Questions (Spanish)**

- ¿Qué sentimientos asocias con el futuro de los arrecifes de coral?
- ¿Qué sentimientos asocia con la restauración de coral?
  - *¿Cómo describirías la experiencia de participar en la restauración de corales a otras personas en tu vida que no la han hecho?*
- En la encuesta, respondiste "Y" en respuesta a "Hago mucho para proteger la salud de los arrecifes de coral". ¿Por qué te diste esa respuesta?
  - *¿Cómo protege la salud de los arrecifes de coral?*
  - *¿Qué acciones para proteger la salud de los arrecifes te gustaría tomar más?*
  - *Para realizar esas acciones más fácilmente, ¿Qué ayudaría a personas como vos?*
- En la encuesta, escribió 'X' como una forma de proteger la salud de los arrecifes de coral. ¿Puedes decirme más acerca de por qué elegiste eso?
  - *Describe lo que significa [X].*
  - *¿Puede decirme cómo/por qué eso protege la salud de los arrecifes?*
  - *¿Te ocurren algunas respuestas adicionales ahora?*
- ¿Cuáles han sido tus aprendizajes principales de [enseñar/participar en] la clase de la teoría de restauración de corales?
- ¿Cuáles han sido tus aprendizajes principales al [enseñar/participar en] los buceos para la restauración de corales?
  - *Si enseña restauración: ¿Qué esperas que sean los aprendizajes principales del curso para los buceadores que enseñas?*
- ¿Hay algo más que te gustaría agregar?
